# Metabolic Syndrome Augments the Risk of Early Neurological Deterioration in Acute Ischemic Stroke Patients Independent of Inflammatory Mediators: A Hospital-Based Prospective Study

**DOI:** 10.1155/2016/8346301

**Published:** 2016-03-29

**Authors:** Xiaohao Zhang, Zhiguang Sun, Caixia Ding, Yinyan Tang, Xuemei Jiang, Yi Xie, Chuanyou Li, Lankun Zhang, Dan Hu, Tingting Li, Gelin Xu, Lei Sheng

**Affiliations:** ^1^Department of Neurology, Second Affiliated Hospital of Nanjing University of Chinese Medicine, Nanjing, Jiangsu 210002, China; ^2^Department of Gastroenterology, Second Affiliated Hospital of Nanjing University of Chinese Medicine, Nanjing, Jiangsu 210002, China; ^3^Department of Neurology, Jinling Hospital, Nanjing University School of Medicine, Nanjing, Jiangsu 210002, China

## Abstract

*Background and Aims*. Metabolic syndrome (MetS) has been associated with occurrence and prognosis of ischemic stroke. This study aimed to evaluate whether an association exists between MetS and early neurological deterioration (END) following acute ischemic stroke and the possible role inflammatory biomarkers play.* Methods and Results*. We conducted a prospective cohort investigation that involved 208 stroke patients within 48 hours from symptom onset. MetS was determined by the modified National Cholesterol Education Program/Adult Treatment Panel III criteria. END was defined as an increase of ⩾1 point in motor power or ⩾2 points in the total National Institutes of Health Stroke Scale (NIHSS) score within 7 days. Univariate logistic regression analysis showed that patients with MetS had a 125% increased risk of END (OR 2.25; 95% CI 1.71–4.86, *P* = 0.005). After adjustment for fibrinogen and high-sensitivity C-reactive protein, MetS remained significantly correlated to END (OR 2.20; 95% CI 1.10–4.04, *P* = 0.026) with a 77% elevated risk per additional MetS trait (OR 1.77; 95% CI 1.23–2.58, *P* = 0.002).* Conclusions*. This study demonstrated that MetS may be a potential predictor for END after ischemic stroke, which was independent of raised inflammatory mediators.

## 1. Introduction

In China, there are 1.5 to 2 million new stroke cases each year, and stroke has been ranked as the first leading cause of mortality and long-term disability [1]. Although most patients tend to improve in the first few days after stroke, a recognized fraction does not virtually recover but deteriorates, which has been termed as early neurological deterioration (END) [2]. Of note, END has been observed in approximately 5%–40% of patients with acute ischemic stroke [2–4]. And END is important for stroke prognosis because it may portend a higher risk of death and increased dependency in daily living [5–7]. Accordingly, it is important to detect and manage factors for END in order to improve stroke outcomes.

Metabolic syndrome (MetS), a constellation of interconnected vascular risk factors, includes insulin resistance/diabetes, elevated blood pressure, central obesity, and dyslipidemia [8]. Over the past two decades, the number of patients with MetS has enlarged strikingly in China [9]. There are a few studies reporting a positive relationship between MetS and unfavorable outcome after ischemic stroke [10, 11]. Nevertheless, whether MetS is a novel risk factor for acute stroke complication, such as END, has not been well addressed. On the other hand, high-sensitivity C-reactive protein (hsCRP) and fibrinogen, which are generally considered biomarkers of low-grade chronic systemic inflammation, have been implicated in the pathophysiology of MetS [12]. Meanwhile, it may be one of the molecular mechanisms involved in ischemic stroke [13]. We further hypothesized that the association of MetS with an increased risk of END may be partially explained by higher levels of fibrinogen and hsCRP. We therefore performed this study to investigate the role of MetS and its components in the prediction of END in a hospital-based prospective study.

## 2. Experimental Methods

### 2.1. Study Populations

We prospectively screened patients in the Second Affiliated Hospital of Nanjing University of Chinese Medicine from Jan 2013 to Sep 2015. Patients with first-ever acute ischemic stroke aged 18 years or older and evaluated within 48 hours of symptom onset were included in the study. Patients with intravenous thrombolysis, history of brain surgery, tumor, presence of severe renal disease and hepatic disease, infectious disease, and early discharge were excluded. All participants consented to participation in the study in accordance with the research ethics attained through local ethics review committees.

### 2.2. Clinical Assessment

Data collection was performed using a standardized case report form. General information, previous medication history (including hypertension, diabetes mellitus, hyperlipidemia, atrial fibrillation, and coronary heart disease), the data of physical examination, clinical characteristics, laboratory data including fasting plasma glucose (FPG), hsCRP, fibrinogen, and imaging results were all recorded. Additional assessments consisting of carotid ultrasonography, transcranial Doppler, magnetic resonance angiography, and digital subtraction angiography were used to evaluate brain-supplying arteries. Cardiac diagnostic tests such as electrocardiography and transthoracic echocardiography were measured to identify cardioembolic stroke. Stroke subtype was classified according to TOAST (Trial of Org 10172 in Acute Stroke Treatment) criteria [14].

### 2.3. Treatment and Clinical Assessment Protocol

Once admitted in the stroke unit, guideline-based treatments, including monoantiplatelet, dual-antiplatelet, and anticoagulation, were performed in all participants immediately [15]. Oral statin supplementation is mandatory in cases without contradictions. Risk factors were managed according to guidelines during hospitalization.

Stroke severity was assessed by a certified neurologist using the National Institutes of Health Stroke Scale (NIHSS) at admission and continued 1–3 times a day for 7 days. In our study, END was defined as an increment of at least one point in motor power or total NIHSS score ⩾2 points deterioration within the first week after admission [16, 17].

### 2.4. Definition of MetS

According to the modified National Cholesterol Education Program/Adult Treatment Panel III (NCEP ATP III) criteria [18], MetS was recognized as the presence of at least three of the following risk components: (i) central obesity (waist circumference ⩾90 cm in men or ⩾80 cm in women); (ii) triglyceride (TG) ⩾1.70 mmol/L; (iii) high-density lipoprotein (HDL) cholesterol < 1.03 mmol/L (male) or < 1.30 mmol/L (female); (iv) elevated blood pressure: systolic blood pressure ⩾130 mmHg, diastolic blood pressure ⩾85 mmHg, or use for antihypertensive drugs; and (v) hyperglycemia: FPG ⩾5.6 mmol/L or need for antihyperglycemic medication.

### 2.5. Statistical Analysis

Statistical analysis was performed using SPSS software, version 17.0 (SPSS Inc., Chicago, IL). Continuous variables were presented as the means (SD) or medians (interquartile range, IQR) and analyzed with a *t*-test or Mann-Whitney *U* test. Categorical variables were expressed as *n* (%) and analyzed with a chi-square test or Fisher's exact test. One-way analysis of variance was used to evaluate the hsCRP and fibrinogen concentrations in different groups of individuals with 0 to 5 metabolic syndrome risk factors. Then, we performed multivariate logistic regression models to calculate odds ratios (OR) and 95% confidence intervals (CI) for the contribution of MetS and its components and the number of MetS components in the prediction of END. To determine whether inflammatory biomarkers may mediate the relationship between MetS and END, fibrinogen and hsCRP levels were added to the adjusted model. All tests were 2-tailed and statistical significance was established at *P* value of less than 0.05.

## 3. Results

Overall, 208 participants with an average age of 66.3 ± 9.2 (from 39 to 88 years old) were enrolled in this study. The mean time from symptom onset to initial evaluation was 24.6 ± 17.1 hours, and the median NIHSS score at admission was 3 points. More than 41% of the cohort met the criteria for MetS. Among patients with MetS, hypertension presented was the most prevalent MetS trait (96.5%), followed by obesity (80.4%), hyperglycemia (80.4%), hypertriglyceridemia (48.3%), and decreased HDL (18.4%). In total, 21.6%, 36.5%, 32.7%, and 9.1% patients had 0-1, 2, 3, and ⩾4 MetS traits, respectively.

MetS was more prevalent in females than males (51.5% versus 37.1%, *P* = 0.049). Notably, median hsCRP levels were 4.0 mg/L and 2.2 mg/L (*P* = 0.039) in patients with and without MetS. Among subjects with 0 to 5 MetS components, median hsCRP levels rose from 1.8 to 4.9 mg/L (*P* = 0.019). However, no association was found between fibrinogen levels and presence of MetS.

During the initial 7 days after admission, 49 patients were identified with END, which accounted for 23.6% [95% CI 20.7%–26.5%] of the cohort.  Table 1 illustrated the baseline characteristics, inflammatory status, and MetS between the subgroups according to the presence or absence of END. Compared with patients without END, those with it were older (68.7 ± 9.8 versus 65.6 ± 8.9 years, *P* = 0.040), developing higher prevalence of diabetes mellitus (46.9% versus 25.8%, *P* = 0.005), MetS (59.2% versus 36.5%, *P* = 0.005), and increased number of MetS components (*P* = 0.026). We obtained the similar results when examining plasma inflammatory biomarkers. Patients with END had higher levels of leukocyte count (7.5 ± 1.5 versus 6.9 ± 1.810^9^/L, *P* = 0.038), hsCRP (6.0 versus 2.0 mg/L, *P* = 0.001), and homocysteine (14.4 versus 11.1 umol/L, *P* = 0.049), with longer hospital stay (18.0 versus 13.0 day, *P* = 0.001).

**Figure 1:**
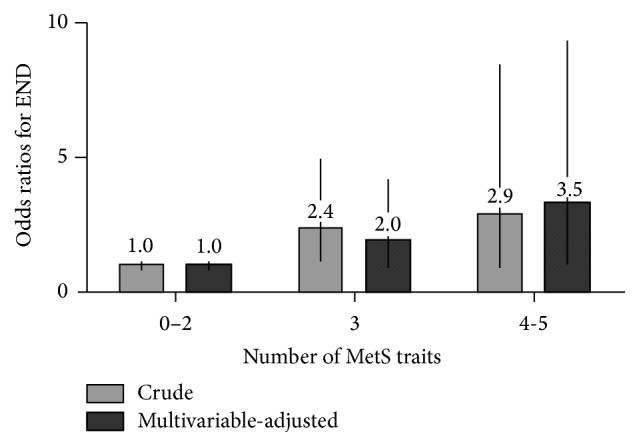
Odds ratios for END according to the number of MetS traits. MetS, metabolic syndrome; END, early neurological deterioration. Data are crude (light bars) and multivariable-adjusted (dark bars) odds ratios. Multivariable model was adjusted for levels of fibrinogen and high-sensitivity C-reactive protein. Error bars represent 95 percent confidence intervals.

Univariate logistic regression analysis revealed that MetS was positively correlated to increasing risk of END in ischemic stroke patients (OR 2.25; 95% CI 1.71–4.86, *P* = 0.005). Only one MetS component, hyperglycemia, was associated with greater END risk (OR 5.83; 95% CI 2.81–12.08, *P* < 0.001). Findings were similar when measured according to the number of MetS traits (Figure 1). After adjusting for age and sex, hyperglycemia (OR 6.33; 95% CI 2.99–13.39, *P* < 0.001) and MetS (OR 3.08; 95% CI 1.54–6.16, *P* = 0.004) were related with END (Model 1, Table 2). This trend remained significant after controlling for levels of fibrinogen and hsCRP (Model 2, Table 2).

## 4. Discussion

Our prospective study found that ischemic stroke patients with MetS were at increasing risk of developing END. We also showed that the risk of END was positively associated with the accumulation of MetS components. Risk relationships were not appreciably attenuated after adjustment for levels of fibrinogen and hsCRP, suggesting that the excess risk with MetS may not be mediated by heightened inflammation.

In contrast to later neurological deterioration which usually results from systemic complications, END is more likely to be related to biochemical abnormality such as hyperglycemia and inflammation [19]. As similar to previous studies [20, 21], our cohort demonstrated 5.8-fold increased odds ratios of END (OR 5.83; 95% CI 2.81–12.08, *P* < 0.001) among those with hyperglycemia. Possible mechanisms of hyperglycemia-associated neurological deterioration could be the fact that it induces endothelial damage, intracellular acidosis, and blood-brain barrier disruption [22]. Also, several observational studies have suggested that inflammation may play a critical role in END [23, 24]. Vila et al. [23] found that interleukin-6 in plasma (21.5 pg/mL; OR 37.7, 95% CI 11.9–118.8) and cerebrospinal fluid (>6.3 pg/mL; OR 13.1, 95% CI 2.2–77.3) were significant factors for early clinical worsening in all ischemic stroke subtypes, independent of initial size and topography. Castellanos et al. [24] performed a secondary analysis of 113 consecutive patients with lacunar infarction and reported that high concentrations of interleukin-6, tumor necrosis factor-*α*, and intercellular adhesion molecule-1 in blood were associated with END and Barthel Index < 85. Moreover, plasma interleukin-10, a well-known anti-inflammatory cytokine, was found to be protective for END on multivariate analysis (OR 0.3, 95% CI 0.1–0.9) [25]. Nevertheless, the precise signals underlying END mediated by inflammation are uncertain but may involve neurotoxicity, particularly in conditions of local hypoxia [26]. Thus, identifying metabolic markers for END will conduce to detection of its potential mechanisms and target therapeutic interventions for prevention.

MetS is a growing public health problem worldwide. The prevalence of MetS has reached 58% in elderly Chinese population and it is projected to increase considerably [27]. Evidently MetS has been reported to augment the risk of stroke (relative risk [RR]: 2.27; 95% CI: 1.80 to 2.85), cardiovascular disease (RR: 2.35; 95% CI: 2.02 to 2.73), and all-cause mortality (RR: 1.58; 95% CI: 1.39 to 1.78) [28]. Also, in a study population of 691 subjects with acute ischemic stroke [11], MetS was independently correlated to a higher modified Rankin Scale score at discharge (OR 1.57; 95% CI 1.13–2.19), which was prominent with more MetS traits after being controlled for other risk factors (*P* = 0.030). To our knowledge, the impact of MetS on acute stroke complications has not been evaluated. Our present study implied that MetS increased the incidence of END approximately 2.3-fold (OR 2.25; 95% CI 1.71–4.86, *P* = 0.005). As shown in previous studies [20, 21, 29], metabolic abnormalities that integrate MetS have been in a close relation with aggravation of acute ischemic stroke. MetS-related alterations comprise impairments in endogenous fibrinolytic capacity, endothelial dysfunction, and a proinflammatory state, all of which may contribute to neurological deterioration [30].

It is worthwhile to mention that, in accordance with prior studies [31–33], our data also confirmed that levels of plasma hsCRP (OR 1.05; 95% CI 1.01–1.09, *P* = 0.015), a marker of inflammation, were strongly correlated to MetS. We also found an increase in plasma levels of hsCRP (from 1.8 mg/L to 4.9 mg/L) per additional MetS component. Furthermore, in other prospective studies, higher levels of circulating hsCRP [34], leukocyte count [35], and interleukin-6 [23, 24] have been found to increase the risk of early neurological worsening. Therefore, one potential explanation for findings reported here is that elevated levels of inflammatory biomarkers may mediate the association of MetS with END, whereas when fibrinogen and hsCRP levels were added to the adjusted model, MetS remained a significant associated factor (OR 2.20; 95% CI 1.10–4.04, *P* = 0.026) for END in our study. This is a novel finding which suggested that the relationship between MetS and END may not be mediated by circulating levels of hsCRP and fibrinogen. It is possible that acute cerebral inflammatory responses caused by local cytokines may contribute to END [36]. Other potentially biological mediators may include adiponectin decrease [37], which exerts inflammatory function and exacerbates insulin resistance and gamma-glutamyl transpeptidase increment that disrupts intracellular homeostasis of oxidative stress [38, 39]. Herein, further study is needed to identify the biological pathway by which MetS promotes END.

Several limitations should be stressed in the present study. Firstly, the study was conducted in one center with small sample size, which may generate sampling bias. Secondly, the conception of END varies among different studies [2, 3, 5–7]. However, the definition in our study has been widely recommended by researchers, because worse outcomes have been demonstrated in patients under this scoring [40]. Finally, data were observational. Relationships reported cannot be proved as causality. These issues should be addressed in future multicenter studies.

In summary, the occurrence of END among patients with acute ischemic stroke in our study was 23.6%, which was consistent with previous data ranging from 5% to 40%. MetS may be predictive of END, especially the higher levels of plasma glucose, while this association may not be mediated by systemic inflammation. Further studies with large sample size are needed to investigate these associations comprehensively. Pathophysiological mechanisms and therapeutic considerations also remain to be determined.

## Figures and Tables

**Table 1:** Comparison of clinical characteristics between patients with and without END.

Characteristics	With END (*n* = 49)	Without END (*n* = 159)	*P* value
Age, year	68.7 ± 9.8	65.6 ± 8.9	0.040
Male (%)	32 (65.3)	108 (67.9)	0.733
Prehistory			
Smoking (%)	14 (28.6)	52 (32.7)	0.587
Drinking habits (%)	15 (30.6)	43 (27.0)	0.626
Hypertension (%)	31 (63.3)	120 (75.5)	0.101
Diabetes (%)	23 (46.9)	41 (25.8)	0.005
Hyperlipidemia (%)	10 (20.4)	28 (17.6)	0.658
Atrial fibrillation (%)	6 (12.2)	12 (7.5)	0.307
Coronary heart disease (%)	6 (12.2)	16 (10.1)	0.664
Previous antiplatelet (%)	4 (8.2)	17 (10.7)	0.607
Previous stain (%)	3 (6.1)	15 (9.4)	0.471
SBP, mmHg	150.2 ± 17.1	148.7 ± 17.8	0.603
DBP, mmHg	85.0 ± 10.6	85.1 ± 11.6	0.971
BMI, kg/m^2^	25.5 ± 1.6	25.8 ± 1.7	0.242
NIHSS, score	3 (2, 5)	3 (2, 4)	0.193
Time from onset to admission, h	24 (12, 24)	24 (8, 24)	0.970
Length of stay, day	18 (15.5, 21.5)	13 (11, 14)	0.001
MetS (%)	29 (59.2)	58 (36.5)	0.005
Number of metabolic factors			0.026
0-1	5 (10.2)	40 (25.2)	
2	15 (30.6)	61 (38.4)	
3	22 (44.8)	46 (28.9)	
4-5	7 (14.3)	12 (7.5)	
Stroke subtype (TOAST)			0.864
LAA	28 (57.1)	90 (56.6)	
CE	5 (10.2)	11 (6.9)	
SVO	14 (28.6)	52 (32.7)	
Others	2 (4.1)	6 (3.8)	
Laboratory data			
Leukocyte count, 10^9^/L	7.5 ± 1.5	6.9 ± 1.8	0.038
Platelet count, 10^9^/L	186 (156, 228)	190 (145, 210)	0.817
Fibrinogen, mg/dL	307.8 ± 98.7	286.6 ± 66.8	0.088
hsCRP, mg/L	6.0 (2.0, 11.5)	2.0 (0.9, 5.0)	0.001
Homocysteine, umol/L	14.4 (12.0, 21.7)	11.1 (13.0, 18.0)	0.049
Fasting plasma glucose, mmol/L	8.0 (5.9, 9.9)	5.4 (5.0, 6.1)	0.001
TC, mmol/L	4.8 ± 1.3	4.5 ± 1.0	0.194
TG, mmol/L	1.3 (1.0, 2.2)	1.3 (1.0, 1.7)	0.307
HDL, mmol/L	1.4 (1.1, 1.6)	1.3 (1.1, 1.5)	0.192
LDL, mmol/L	2.3 (1.7, 2.9)	2.3 (1.9, 2.9)	0.750

MetS, metabolic syndrome; END, early neurological deterioration; SBP, systolic blood pressure; DBP, diastolic blood pressure; TC, total cholesterol; TG, triglyceride; HDL, high-density lipoprotein; LDL, low-density lipoprotein; hsCRP, high-sensitivity C-reactive protein; LAA, large artery atherosclerosis; SVO, small vessel occlusion; CE, cardioembolism.

**Table 2:** Logistic regression analysis for the association of MetS and its components with END in ischemic stroke patients.

	Univariate analysis	Model 1	Model 2
	OR (95% CI)	OR (95% CI)	OR (95% CI)
Central obesity	0.98 (0.51–1.88)	1.04 (0.52–2.10)	0.84 (0.42–1.68)
Hyperglycemia	5.83 (2.81–12.08)^b^	6.33 (2.99–13.39)^b^	5.259 (2.49–11.12)^b^
Elevated blood pressure	0.79 (0.34–1.84)	0.76 (0.32–1.78)	0.86 (0.35–2.08)
Decreased HDL	0.92 (0.35–2.42)	1.04 (0.38–2.86)	0.92 (0.31–2.75)
Hypertriglyceridemia	1.69 (0.85–3.38)	1.98 (0.96–4.06)	1.83 (0.88–3.82)
MetS	2.25 (1.71–4.86)^a^	3.08 (1.54–6.16)^a^	2.20 (1.10–4.04)^a^

MetS, metabolic syndrome; END, early neurological deterioration; HDL, high-density lipoprotein; OR, odds ratios; CI, confidence interval; ^a^ *P* < 0.05; ^b^ *P* < 0.001; Model 1 adjusted for age and gender; Model 2 adjusted for levels of fibrinogen and hsCRP.
